# Musical Expertise Reshapes Cross-Domain Semantic Integration: ERP Evidence from Language and Music Processing

**DOI:** 10.3390/brainsci15040401

**Published:** 2025-04-16

**Authors:** Xing Wang, Tao Zeng

**Affiliations:** 1 Department of English, College of Foreign Languages, Hunan University, Changsha 410082, China; wangxing007@hnu.edu.cn; 2 Hunan Provincial Research Centre for Language and Cognition, Changsha 410082, China

**Keywords:** semantic processing, musical expertise, event-related potentials (ERP), N400, P600

## Abstract

**Background/Objectives:** Both language and music are capable of encoding and communicating semantic concepts, suggesting a potential overlap in neurocognitive mechanisms. Moreover, music training not only enhances domain-specific musical processing but also facilitates cross-domain language processing. However, existing research has predominantly focused on Indo-European languages, with limited evidence from paratactic languages such as Mandarin Chinese. In addition, the impact of variations in musical expertise on these shared processing mechanisms remains unclear, leaving a critical gap in our understanding of the shared neural bases for semantic processing in language and music. This event-related potential (ERP) study investigated whether Chinese sentences and musical chord sequences share semantic processing mechanisms and how musical expertise modulates these mechanisms. **Methods:** This study recruited 46 college students (22 musicians and 24 non-musicians). Participants read Chinese sentences presented word-by-word visually, while chord sequences were delivered auditorily, with each word temporally aligned to one chord. Sentences included semantically acceptable or unacceptable classifier–noun pairs and chord sequences ended with in-key or out-of-key chords. Participants were instructed to focus on reading sentences while ignoring the concurrent music. ERP signals were recorded, and time-locked to final words to capture neural dynamics during semantic integration. **Results:** The behavioral results showed that musicians were influenced by musical regularity when reading (acceptable: *F*(1, 44) = 25.70, *p* < 0.001, *η_p_*^2^ = 0.38; unacceptable: *F*(1, 44) = 11.45, *p* = 0.002, *η_p_*^2^ = 0.21), but such effect was absent in non-musicians (*p*s > 0.05). ERP results showed that musical semantic processing had a substantial impact on both P200 (*F*(1, 44) = 9.95, *p* = 0.003, *η_p_*^2^ = 0.18), N400 (musicians: *F*(1, 44) = 15.80, *p* < 0.001, *η_p_*^2^ = 0.26; non-musicians: *F*(1, 44) = 4.34, *p* = 0.043, *η_p_*^2^ = 0.09), and P600 (musicians: *F*(1, 44) = 5.55, *p* = 0.023, *η_p_*^2^ = 0.11; non-musicians: *F*(1, 44) = 8.68, *p* = 0.005, *η_p_*^2^ = 0.17) components. Furthermore, musical expertise exerted modulatory effects during later stages, as evidenced by divergent N400 and P600 latency patterns between musicians and non-musicians. Specifically, ERP amplitudes exhibited opposing trends: musicians showed an enhanced N400 and diminished P600, while non-musicians displayed a weaker N400 and stronger P600. **Conclusions:** Our findings provide novel evidence that Mandarin Chinese and chord sequences engage partially overlapping neural mechanisms for semantic processing both in the early (P200) and the late (N400 and P600) stages. Crucially, this study is the first to demonstrate that musical expertise may gradually reorganize these shared mechanisms, enabling two initially independent but functionally analogous semantic mechanisms into a domain-general processing system. These insights deepen our understanding of the neurocognitive mechanisms underlying linguistic and musical semantic processing and highlight how expertise shapes the neural architecture of cross-domain mechanisms.

## 1. Introduction

Language and music are two unique human cognitive abilities [1]. Both are hierarchically structured systems, composed of discrete elements governed by combinatorial rules [2,3], and existing evidence has suggested that the neural substrates involved in syntactic processing in language and music partially overlap [4,5,6,7]. Beyond these syntactic parallels, language and music have the remarkable ability to encode and communicate semantic concepts [8,9]. However, whether these two domains share semantic processing mechanisms remains debated. Existing research has focused on Indo-European languages, leaving a critical gap in understanding semantic processing commonalities between music and paratactic languages such as Chinese, where processing relies mainly on the lexical and contextual meaning of each word. Critically, advanced musical expertise enhances not only music-specific processing [10,11] but also provides cross-domain benefits to linguistic abilities [12,13]. This raises the possibility that musical expertise might modulate shared neurocognitive mechanisms between language and music, a hypothesis that warrants systematic investigation. To address these gaps, the present study aims to take a preliminary step to explore the shared semantic processing mechanism between Chinese sentences and music, as well as the potential modulating effect of musical expertise on such a mechanism.

### 1.1. Language Semantics and Music Meaning

The current scholarship identifies three principles of musical meaning: extra-musical meaning, intra-musical meaning, and musicogenic meaning. The first two arise from the cognitive interpretation of musical information, while musicogenic meaning pertains to the physical, emotional, or self-related effects induced by music [14]. Extra-musical meaning arises from the interpretation of musical information in relation to the external world, serving as a bridge between an individual’s auditory perception and external reality [15]. This includes emotions such as happiness or sadness evoked by a specific musical excerpt or the communication of philosophical ideas through music. Intra-musical meaning refers to the significance derived from the interaction of musical elements within a music piece [16,17]. When listening to music, audiences develop expectations about upcoming notes or chords. The extent to which these expectations are met—specifically, the harmonic proximity between the expected and actual notes or chords—determines the listener’s perception of tension and resolution, a process known as the “tension-resolution pattern” [18]. Music psychologists attribute this interplay between expectation and realization to the intrinsic structural properties of music, which reflect the cognitive processing of intra-musical meaning [14,17]. For instance, two notes or chords within the same key are perceived as more closely related than those from different keys. Moreover, within a given key, the tonic or tonic chord is perceived as more stable or final-sounding compared to other elements, as explained by the circle of fifths [19].

Accumulating evidence demonstrates that musical meaning engages neurocognitive mechanisms similar to those underpinning linguistic semantics. First, violations of intra-musical meaning elicit ERP components analogous to those observed in response to semantic violations in linguistic contexts. In language, semantically acceptable words evoke an N400 component, which typically emerges around 200–300 ms after the onset of a semantically incongruent word, peaking at approximately 400 ms. This response indexes either lexical-semantic retrieval demands [20] or the computational costs of semantic integration [21]. Similarly, in music, harmonically unexpected notes or chords evoke a homologous response: harmonically incongruent chords elicit an N500 component approximately 450–1500 ms after stimulus onset, reflecting the difficulty in establishing harmonic context or integrating harmonic semantics [14]. Furthermore, experiments have also identified bidirectional cross-domain semantic priming effects between language and music. Whether language primes music or vice versa, the N400 amplitude is significantly reduced in semantically related conditions compared to unrelated scenarios [9,22,23,24,25]. These findings collectively suggest that language and music may engage overlapping neural mechanisms during semantic processing.

Building on these findings, researchers have further explored the overlap between linguistic and musical semantic processing by examining potential interference effects through the simultaneous presentation of chord sequences and sentences. Poulin-Charronnat et al. [26] conducted a behavioral study that manipulated both the semantic predictability of sentence-final words in French sentences and the harmonic regularity of final chords in chord sequences. Their findings revealed that semantic prediction effects were amplified when highly predictable words were paired with harmonically regular chords, a phenomenon stemming from cross-domain resource competition. This behavioral interference pattern directly supports the hypothesis of overlapping mechanisms for semantic integration, suggesting a shared cognitive architecture for meaning processing in both language and music. Building on this, Perruchet and Poulin-Charronnat [27] revealed that harmonic violations (out-of-key chords) significantly prolonged reading times for syntactically ambiguous English garden-path sentences compared to in-key conditions. This behavioral interface effect further supports the hypothesis of overlapping semantic resources in language and music despite the absence of overt linguistic semantic violations. Neuropsychological studies provide additional support for this claim. Steinbeis and Koelsch [18] pioneered the use of a simultaneous presentation paradigm in an ERP study to probe semantic sharing between language and music. They found that the processing of intra-musical meaning (e.g., harmonic violations) was influenced by concurrent sentence-level semantic processing. Specifically, the N500 component exhibited reduced amplitude in response to out-of-key chords when paired with semantically incongruent sentences, compared to congruent ones. This suggests competition for shared neural resources between language and music during semantic context establishment.

As a paratactic language with minimal morphosyntactic inflections [28], Chinese relies more heavily on semantic information during sentence comprehension [29]. This results in distinct patterns of semantic resource sharing compared to Indo-European languages. Recently, Wang et al. [30] extended this research by applying the simultaneous presentation paradigm to Chinese sentences and chord sequences. They identified an interaction effect between language and music during the N400 phase, providing evidence that cross-domain semantic resource sharing occurs regardless of language type.

While these studies collectively support the idea of partial neural overlaps in semantic integration, several critical questions remain unresolved. Existing research has focused predominantly on late-stage semantic processing (N400/N500 time windows), while relatively few studies have investigated whether Chinese and music share neurocognitive resources during the early stage. This gap leaves early perceptual-cognitive interactions largely unexplored, highlighting the need for further investigation.

### 1.2. A Potential Modulator: Cognitive Control

If two domains share processing resources, alterations in one domain could induce corresponding changes in the shared neural system [31]. This implies that the shared processing mechanism is not fixed but may instead be modulated by certain factors, such as musical expertise. Emerging research indicates that language and music may rely on overlapping reintegration processes associated with cognitive control [32]. Reintegration refers to the process of revising current understanding when new information conflicts with prior knowledge, followed by the construction of a new coherent interpretation. This process of conflict detection and resolution is a hallmark of cognitive control, requiring dynamic regulation of neural activity and rapid adaptive adjustments in response to competing stimuli [33,34].

Music processing involves not only reintegration but also the engagement of cognitive control. As music unfolds, the brain dynamically constructs cognitive representations of both structural and semantic features. This process involves not only the integration of musical elements but also the anticipation of upcoming elements [35]. Empirical evidence highlights the critical role of cognitive control in music processing. For example, in a study where participants performed a Stroop task while being exposed to regular or irregular chord sequences, harmonic expectations were found to interact with Stroop interference effects. This interaction suggests that cognitive control is a fundamental mechanism underlying music processing [36].

Existing research has shown that musical training enhances cognitive control abilities [11,37], suggesting that it may modulate the shared processing mechanism between language and music. Playing musical instruments engages multiple brain regions and recruits high-level cognitive systems to ensure fluent performance [38,39]. Compared to non-musicians, individuals with long-term musical training exhibit superior cognitive control, including enhanced conflict monitoring, reduced Stroop interference [40,41], and shorter latencies with greater amplitudes in the P300 component, which reflects cognitive control ability [42,43,44]. These findings collectively indicate that music training enhances not only domain-specific musical processing abilities but also domain-general cognitive control abilities. This aligns with the “Unified Theory of Performance” [45], which proposes that music training, as a high-level cognitive processing skill, promotes broader cognitive development. Furthermore, structural neuroplasticity further supports this relationship: music training has been shown to increase gray matter density in auditory regions, the corpus callosum, multimodal integration brain regions, and the inferior frontal gyrus, which, in turn, improves the brain’s information transmission, language processing, executive functions, and working memory capacities [46,47]. In sum, these findings suggest that musical expertise may act as a potential modulator of the shared processing mechanisms between language and music.

### 1.3. The Present Study

Existing research converges on the notion that language and music may share neural mechanisms for semantic processing. However, critical gaps in the literature remain.

First, studies investigating cross-domain shared semantic mechanisms between language and music have been confined to Perruchet and Poulin-Charronnat [27], Poulin-Charronnat et al. [26], Steinbeis and Koelsch [18], and Wang et al. [30]. The first two studies relied on behavioral methods, which lack the temporal resolution to disentangle neural interactions between the two domains. Second, most studies have focused on Indo-European languages, raising questions about the generalizability of their findings, especially in the case of Mandarin Chinese, which places a greater emphasis on semantic processing [48]. Although Wang et al. [30] examined shared semantic processing mechanisms between Mandarin Chinese and music, their research concentrated solely on late controlled stages, overlooking the earlier automatic processing stage. Finally, while musical expertise has been proposed as a potential modulating factor on shared processing mechanisms, empirical evidence remains limited. This leaves an open question: Does musical expertise enhance cross-domain resource sharing between language and music, resulting in distinct neural encoding patterns between musicians and non-musicians?

Against this backdrop, the present study employed the ERP method, drawing on the simultaneous presentation paradigm used by Steinbeis and Koelsch [18] and Wang et al. [30], aiming to investigate the following questions:

- Do Mandarin Chinese sentences and chord sequences share neurocognitive mechanisms for semantic processing, and if so, how do these mechanisms operate?
- How does musical expertise modulate this underlying shared mechanism across both early and late processing stages?

## 2. Methods

### 2.1. Participants

A total of 46 participants were recruited via flyer advertisements and divided into two groups based on musical expertise: musicians and non-musicians. Musical expertise was assessed via a self-reported questionnaire evaluating their formal musical training backgrounds. The musician group consisted of 22 music major college students (12 female) with over eight years of formal instrumental (e.g., piano, violin, or Chinese zither) learning experience [13]. All musicians started their training before the age of 12 and maintained a practice schedule of at least three sessions per week, each lasting more than two hours during the six months preceding the experiment. The non-musician group included 24 non-music major college students (12 female) with no extracurricular musical training. The two groups were carefully matched for age, years of education, native dialect background (all participants were from northern China), and long-term vocabulary knowledge level, assessed by the vocabulary subtest of the WAIS-IV test [49].

All participants were right-handed, had normal or corrected-to-normal vision, and self-reported no history of psychological or neurological disorders. Amusia was ruled out using the Montreal Battery of Evaluation of Amusia (MBEA) [50] with all participants scoring within the normal range across its six subtests. Participants received compensation after completing the experiment. The demographic characteristics of participants and the values of these measures were reported in Table 1.

### 2.2. Stimuli

The experimental stimuli consisted of linguistic and musical materials, adapted from Experiment 2 of Wang et al. [30]. The linguistic stimuli included 60 Chinese sentences, comprising 30 semantically acceptable and 30 unacceptable sentences. All sentences were structured as “subject + verb + numeral + classifier + object-gap relative clause (subject + verb + DE + head noun)” [e.g., “警察捡到了一部游客丢失的手机” (jingcha jiandao le yi bu youke diushi de shouji in Chinese pinyin, meaning “The policeman picked up a cell phone, which might have been left by a tourist.”)]. In acceptable sentences, classifiers were semantically matched with their head nouns (e.g., [“一部手机” (yi bu shouji in Chinese pinyin, meaning “one-CL_classifying electric appliance_ cell phone”)]). In unacceptable sentences, semantic incongruency arose solely from a classifier–noun mismatch (e.g., [“一部钱包” (yi bu qianbao in Chinese pinyin, meaning “one-CL_classifying electric appliance_ wallet”)]). Removal of the number-classifier phrase preserved semantic plausibility [e.g., “警察捡到了游客丢失的手机” (jingcha jiandao le youke diushi de shouji in Chinese pinyin, meaning “The policeman picked up a cell phone, which might have been left by a tourist.”). To ensure the plausibility of the experimental materials, a pretest was conducted, in which 30 participants rated the semantic acceptability of the sentences on a 7-point Likert scale (1 = lowest acceptability, 7 = highest acceptability). A *t*-test comparing acceptable and unacceptable sentences revealed significantly lower acceptability ratings for unacceptable sentences (*M* = 1.90, *SD* = 0.76) compared to acceptable sentences (*M* = 6.03, *SD* = 0.81; *t*(58) = 20.41, *p* < 0.001, *d* = 5.27). The acceptable and unacceptable sentences are provided as examples in Table 2.

**Figure 1:**
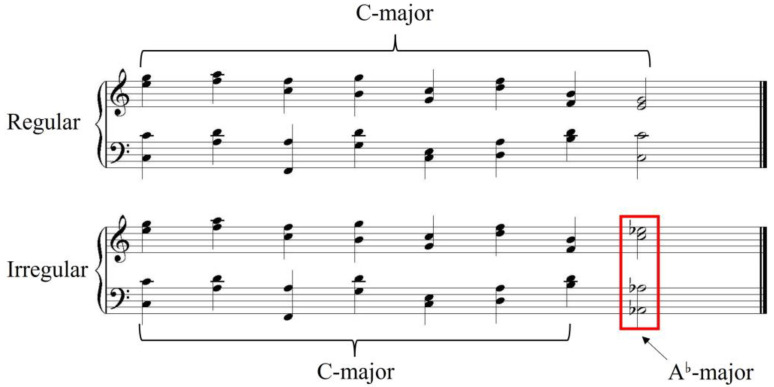
A sample music item in two versions. The first seven chords are in C-major, the top sequence ends in a regular chord (tonic in C-major), and the lower sequence ends in an irregular chord (tonic in A^♭^-major).

The musical stimuli consisted of 30 regular and 30 irregular chord sequences, evenly distributed across three keys: C-, G-, and E-major. Regular sequences followed a paradigmatic cadential structure, concluding with a tonic chord in an authentic cadence. Irregular sequences substituted the final tonic chord with a non-diatonic chord, created by shifting the original chord more than three positions away from the tonic on the circle of fifths (e.g., replacing a C-major tonic with A^♭^. major in a C-major context), thereby disrupting tonal resolution (see Figure 1). To ensure unambiguous classification of chord sequences, all stimuli underwent a binary acceptability test (0 = irregular, 1 = regular) administered to 30 non-musicians. The results indicated perfect accuracy: All participants correctly identified regular and irregular sequences (100% correct discrimination). 

### 2.3. Procedure

Participants were seated in a sound-attenuated, electrically shielded room facing a computer monitor. An EEG cap was fitted to record their neural activity during the experiment, and they were instructed to minimize body movements to reduce artifacts. Sentences were presented visually word by word, alongside aurally played musical sequences, with each word synchronized to a corresponding chord. Each trial began with a 500 ms fixation cross, followed by sequential word–chord pairs. Each pair was presented for 600 ms, except the final pair, which lasted 1200 ms [7,18]. Participants were informed that the sentences and chord sequences would be presented simultaneously, However, they were only notified about variations in sentence types and were instructed to focus exclusively on the screen and judge the semantic congruency by pressing buttons with the first fingers of their right and left hand after each trial. The concurrent chord sequences were explicitly designated as task-irrelevant stimuli, and participants were instructed to ignore them [7,30]. Response-button mappings were counterbalanced across participants [51]. The order of trial presentation was pseudorandomized to ensure that no more than three consecutive sentences belonged to the same condition.

### 2.4. EEG Recording and Preprocessing

The EEG signals were recorded by a 64-channel Ag/AgCl electrode Quick-Cap (International 10–20 system) in DC mode using NeuroScan Acquire Software with a SynAmps2 amplifier (Version 8.0.5.0, Compumedics, Melbourne, Australia). The VEOG and HEOG signals were recorded via four additional electrodes. Signals were sampled at 1000 Hz, with the nose tip as a physical reference. The electrode between Fz and Cz was selected as the ground, and all electrode impedance levels were kept below 5 kΩ.

Preprocessing was conducted using MATLAB 2024a (MathWorks), the EEGLAB toolbox 2024.2 [52], and the Evoked ERP_ERO_v1.1 toolbox [53]. Continuous EEG signals were referenced to the average of the left and right mastoids, with a bandpass filter of 0.1–30 Hz. Independent component analysis (ICA) was applied to isolate and remove components presenting ocular (e.g., blinks, saccades) and other noise sources. Epochs were extracted from 200 ms pre-stimulus to 1000 ms post-stimulus onset to the final words of each sentence. Trials with artifacts exceeding an amplitude of ±100 μV in any channel were rejected, resulting in the exclusion of approximately 8.15% of trials across participants.

### 2.5. Data Analysis

All data were analyzed by calculating mean amplitudes for each condition. ERPs were averaged offline across epochs from 200 ms pre-stimulus to 1000 ms post-stimulus onset for final words, with a 200 ms pre-stimulus baseline correction. Based on a visual inspection of the grand average waveform and previous findings on P200, N400, and P600 components [30], the time windows of 150–250 ms, 300–450 ms, and 500–700 ms after target-stimulus onset were selected for statistical analysis. The electrodes were grouped into 9 regions of interest (ROI): left anterior (F1, F3, F5, FC1, FC3, FC5), left central (C1, C3, C5, CP1, CP3, CP5), left posterior (P1, P3, P5, PO3, PO5), middle anterior (Fz, FCz), middle central (Cz, CPz), middle posterior (Pz, POz), right anterior (F2, F4, F6, FC2, FC4, FC6), right central (C2, C4, C6, CP2, CP4, CP6), and right posterior (P2, P4, P6, PO4, PO6). ERP effects were analyzed separately for midline and lateral sites. For midline electrodes, repeated-measures ANOVAs were conducted on average ERP amplitudes, with the following within-subjects factors: language (semantically acceptable, semantically unacceptable), music (regular, irregular), and region (anterior, central, posterior). For lateral sites, the within-subject factors included language, music, region, and hemisphere (left, right). The group (musicians, non-musicians) was treated as a between-subjects factor. Significant interactions between experimental variables were further examined by simple effects tests. The Greenhouse–Geisser correction was applied when evaluating effects with more than one degree of freedom in the numerator. Statistical significance was defined as a two-tailed *p*-value < 0.05. Values within the 0.05–0.1 range were designated as marginally significant.

## 3. Results

### 3.1. Behavioral Results

**Figure 2:**
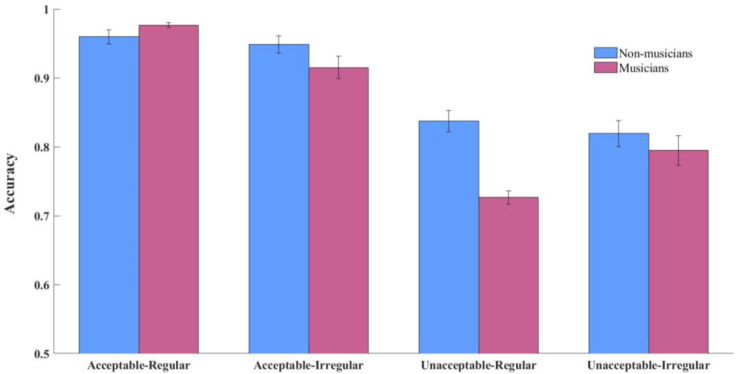
Accuracy rates across conditions for non-musicians and musicians.

Both musicians and non-musicians demonstrated high task engagement, as evidenced by their mean accuracy rates on comprehension questions (see Figure 2), which exceeded the chance level across all conditions (all *p*s < 0.001). A repeated-measures ANOVA revealed a significant three-way interaction among language, music, and group (*F*(1, 44) = 15.55, *p* < 0.001, *η_p_*^2^ = 0.27). Simple effect analysis for language showed that both groups performed better on semantically acceptable sentences compared to unacceptable ones across both musical conditions (non-musicians: *F*_Regular_(1, 44) = 58.33, *p* < 0.001, *η_p_*^2^ = 0.58, *F*_Irregular_(1, 44) = 20.16, *p* < 0.001, *η_p_*^2^ = 0.32; musicians: *F*_Regular_(1, 44) = 203.37, *p* < 0.001, *η_p_*^2^ = 0.83, *F*_Irregular_(1, 44) = 14.50, *p* < 0.001, *η_p_*^2^ = 0.26). Simple effect analysis for music revealed that musicians exhibited enhanced performance on regular sequences than irregular sequences when processing unacceptable sentences (*F*(1, 44) = 25.70, *p* < 0.001, *η_p_*^2^ = 0.38). Unacceptable sentences were judged better when paired with irregular sequences than with regular sequences (*F*(1, 44) = 11.45, *p* = 0.002, *η_p_*^2^ = 0.21). Non-musicians, however, displayed no significant music-related performance differences across sentence types (acceptable: *F*(1, 44) = 1.00, *p* = 0.323, *η_p_*^2^ = 0.02; unacceptable: *F*(1, 44) = 0.96, *p* = 0.333, *η_p_*^2^ = 0.02).

### 3.2. ERP Results

**Figure 3:**
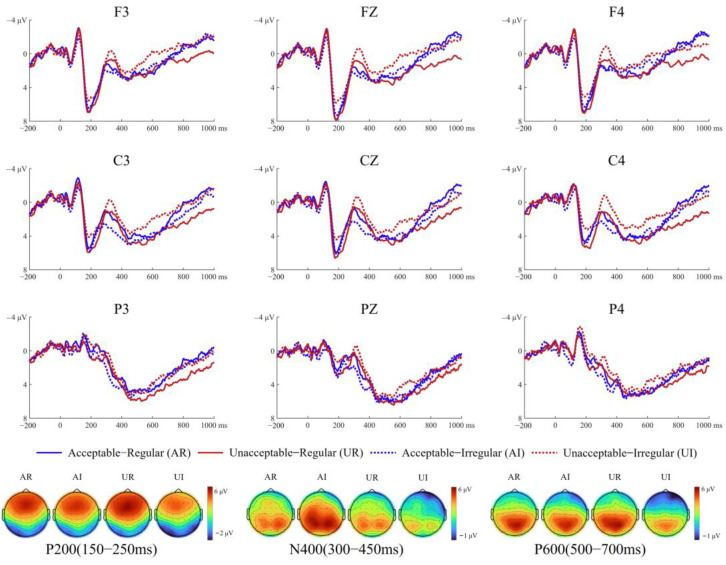
Grand-average ERP waveforms for non-musicians. Grand-average time courses at final words across four experimental conditions from nine representative electrodes. Scalp topographical maps show the spatial distribution of the P200, N400, and P600 components for each condition, averaged over 150–250 ms, 300–450 ms, and 500–700 ms time windows, respectively.

**Figure 4:**
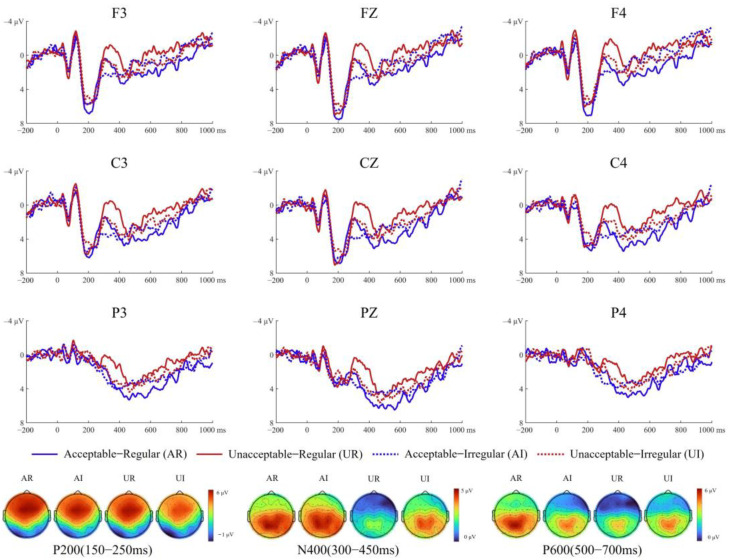
Grand-average ERP waveforms for musicians. Grand-average time courses at the final words across four experimental conditions from nine representative electrodes. Scalp topographical maps show the spatial distribution of the P200, N400, and P600 components for each condition, averaged over 150–250 ms, 300–450 ms, and 500–700 ms time windows, respectively.

Figure 3 and Figure 4 show the average ERP waveforms and corresponding topographic maps for non-musicians and musicians, respectively, under the four experimental conditions.

#### 3.2.1. P200

Midline A two-way interaction between music and anteriority was significant (*F*(2, 88) = 5.08, *p* = 0.022, *η_p_*^2^ = 0.10). Simple effect analysis for music revealed that irregular chords elicited more negative amplitudes than regular chords across anterior (*F*(1, 44) = 11.66, *p* = 0.001, *η_p_*^2^ = 0.21) and central (*F*(1, 44) = 12.51, *p* = 0.001, *η_p_*^2^ = 0.22) regions. Another significant two-way interaction between language and music was observed (*F*(1, 44) = 5.75, *p* = 0.021, *η_p_*^2^ = 0.12). Simple effect analysis for language revealed that acceptable sentences elicited larger amplitudes than unacceptable sentences when paired with irregular chords (*F*(1, 44) = 14.16, *p* < 0.001, *η_p_*^2^ = 0.24). Simple effect analysis for music revealed that regular chords elicited larger amplitudes than irregular chords when paired with unacceptable sentences (*F*(1, 44) = 9.95, *p* = 0.003, *η_p_*^2^ = 0.18).

Lateral sides A significant three-way interaction was found among music, anteriority, and hemisphere (*F*(2, 88) = 5.72, *p* = 0.010, *η_p_*^2^ = 0.12). Simple effect analysis for music revealed that regular chords elicited larger amplitudes than irregular chords across both anterior (left: *F*(1, 44) = 8.25, *p* = 0.006, *η_p_*^2^ = 0.16; right: *F*(1, 44) = 14.08, *p* = 0.001, *η_p_*^2^ = 0.24) and central regions (left: *F*(1, 44) = 11.50, *p* = 0.001, *η_p_*^2^ = 0.21; *F*(1, 44) = 10.96, *p* = 0.002, *η_p_*^2^ = 0.20). Simple effect analysis for hemisphere demonstrated that under both regular (*F*(1, 44) = 4.52, *p* = 0.029, *η_p_*^2^ = 0.09) and irregular (*F*(1, 44) = 4.18, *p* = 0.047, *η_p_*^2^ = 0.09) conditions, amplitudes were significantly larger in the left hemisphere than in the right hemisphere. Additionally, a significant two-way interaction between language and music was observed (*F*(1, 44) = 4.88, *p* = 0.032, *η_p_*^2^ = 0.10). Simple effect analysis for language indicated that acceptable sentences elicited larger amplitudes than unacceptable sentences when paired with irregular chords (*F*(1, 44) = 9.60, *p* = 0.003, *η_p_*^2^ = 0.18). Conversely, simple effect analysis for music showed that regular chords elicited larger amplitudes than irregular chords when paired with unacceptable sentences (*F*(1, 44) = 12.92, *p* = 0.001, *η_p_*^2^ = 0.23).

#### 3.2.2. N400

Midline A significant three-way interaction among language, music, and group was observed (*F*(1, 44) = 9.55, *p* = 0.003, *η_p_*^2^ = 0.18). Simple effect analysis for language revealed that in non-musicians, unacceptable sentences elicited larger N400 amplitudes than acceptable sentences when paired with irregular chords (*F*(1, 44) = 13.98, *p* = 0.001, *η_p_*^2^ = 0.24). In contrast, in musicians, this N400 effect was significant when paired with regular chords (*F*(1, 44) = 25.06, *p* < 0.001., *η_p_*^2^ = 0.36). Simple effect tests for music indicated that in non-musicians, irregular chords marginally enhanced N400 amplitudes compared to regular chords paired with incongruent sentences (*F*(1, 44) = 3.43, *p* = 0.071, *η_p_^2^* = 0.07), while in musicians, irregular chords reduced N400 amplitudes for unacceptable sentences (*F*(1, 44) = 13.01, *p* = 0.001, *η_p_*^2^ = 0.23).

Lateral sites A significant three-way interaction among language, music, and group was observed (*F*(1, 44) = 10.48, *p* = 0.002, *η_p_*^2^ = 0.19). Simple effect analysis for language revealed that in non-musicians, unacceptable sentences elicited larger N400 amplitudes than acceptable sentences when accompanied by irregular chords (*F*(1, 44) = 14.05, *p* = 0.001, *η_p_*^2^ = 0.24). In musicians, unacceptable sentences elicited larger N400 amplitudes than acceptable sentences when regular chords were played (*F*(1, 44) = 30.81, *p* < 0.001, *η_p_*^2^ = 0.41). Simple effect analysis for music showed that in non-musicians, irregular chords enhanced the N400 amplitude for unacceptable sentences, whereas in musicians, irregular chords reduced the N400 amplitude for unacceptable sentences.

#### 3.2.3. P600

Midline A three-way interaction among language, music, and group was significant (*F*(1, 44) = 7.38, *p* = 0.009, *η_p_*^2^ = 0.14). Simple effect analysis for language showed that in musicians, acceptable sentences evoked larger P600 amplitudes than unacceptable sentences when paired with regular chords (*F*(1, 44) = 9.50, *p* = 0.004, *η_p_*^2^ = 0.18). In contrast, no significant differences were found between language conditions in non-musicians (regular: *F*(1, 44) = 0.07, *p* = 0.794, *η_p_*^2^ = 0.01; irregular: *F*(1, 44) = 2.67, *p* = 0.110, *η_p_*^2^ = 0.03). Simple effect analysis for music revealed that in non-musicians, irregular chords decreased P600 amplitudes compared to regular chords for unacceptable sentences (*F*(1, 44) = 8.14, *p* = 0.007, *η_p_*^2^ = 0.16). However, no significant effects were observed in musicians (acceptable: *F*(1, 44) = 2.52, *p* = 0.120, *η_p_*^2^ = 0.05; irregular: *F*(1, 44) = 1.79, *p* = 0.188, *η_p_*^2^ = 0.03).

Lateral site A significant three-way interaction among language, music, and group was found (*F*(1, 44) = 8.27, *p* = 0.006, *η_p_*^2^ = 0.16). Simple effect analysis for language showed that in non-musicians, congruent sentences evoked marginally larger P600 amplitudes than incongruent sentences when paired with irregular chords (*F*(1, 44) = 3.80, *p* = 0.058, *η_p_^2^* = 0.08). In musicians, acceptable sentences elicited significantly larger P600 amplitudes than unacceptable sentences when paired with regular chords (*F*(1, 44) = 16.05, *p* < 0.001, *η_p_*^2^ = 0.27).

## 4. Discussion

The current study investigated whether Mandarin Chinese sentences and chord sequences engage overlapping neural mechanisms during simultaneous processing, and how musical expertise modulates this shared mechanism. A stimulus set, specially designed to include sentence-chord sequence pairs, was employed to examine two groups of college students differing in musical expertise (musicians and non-musicians). ERPs were recorded during online sentence comprehension, time-locked to the head nouns of classifiers at the sentence-final position. Behavioral results confirmed that all participants completed the sentence comprehension task despite interference from simultaneously presented task-irrelevant chord sequences. ERP analyses demonstrated distinct patterns of shared semantic processing between musicians and non-musicians, with nuanced group differences in neural engagement. Specifically, during the early processing stage, both groups exhibited P200 effects in response to semantically unacceptable sentences, with amplitudes modulated by musical regularity. This indicates automatic cross-domain interactions between Mandarin Chinese and chord sequences. However, no early-stage influence of musical expertise was observed. At the late stage, semantically unacceptable sentences elicited N400 and P600 components. The amplitudes of these components were similarly influenced by musical regularity, further supporting shared semantic mechanisms across domains [18,30]. Critically, group differences emerged in how musical regularity modulates N400 and P600 responses. Non-musicians showed enhanced N400 amplitudes for semantically unacceptable nouns but attenuated P600 amplitudes for semantic nouns paired with irregular chords. In contrast, musicians exhibited reduced N400 amplitudes and increased P600 amplitudes under the same irregular chord conditions. These findings suggest that musical expertise may selectively shape the neurocognitive dynamics of shared semantic processing between language and music. In conclusion, the results demonstrate engaging overlapping semantic processing mechanisms between Chinese and music across early (automatic) and late (controlled) stages. Musical expertise acts as a key modulator of this shared system, driving divergent neurocognitive patterns between groups. These findings highlight the interplay between domain-general and experience-dependent factors in language-music interactions.

### 4.1. The Underlying Shared Mechanism at the Early Stage

The ERP results revealed a positive-going potential within the 150–250 ms time window, with reduced amplitudes elicited by semantically unacceptable sentences compared to acceptable ones, suggesting that both groups detected semantic anomalies at this stage. This fronto-central distributed positivity is reminiscent of the P200 component, a well-established electrophysiological marker of early semantic integration [54,55,56], which is typically sensitive to contextual expectations in visual paradigms, where larger amplitudes reflect anticipated lexical input [57]. Notably, eye-movement studies have identified semantic integration processes around 250 ms, as evidenced by prolonged first fixation durations for semantically unacceptable sentences compared to acceptable ones at critical regions [58]. In sentence processing, classifiers strongly constrain expectations for subsequent head nouns. When participants encountered the classifier, they likely generated robust predictions for a semantically appropriate noun. Congruent nouns aligned with predictions, eliciting larger P200 amplitudes, whereas incongruent nouns violated these expectations, resulting in attenuated amplitudes. These findings align with those of Zhang et al. [56] who observed reduced P200 amplitudes for semantically incongruent words during Chinese reading, further supporting its role in rapid, transient integration. Importantly, the P200 positivity was not modulated by musical expertise, suggesting that early cross-domain semantic interactions between language and music occur automatically, independent of specialized musical training.

An alternative explanation posits the P200 component as an index of selective attention allocation [59]. According to this view, a reduced P200 amplitude indicates heightened attentional resource demand during subsequent processing stages. When low-probability or unexpected words are encountered, increased cognitive load arises during early perceptual matching due to the need for additional attentional resources [60,61]. In this study, unexpected nouns conflict with participants’ preactivated contextual expectations, requiring more attentional resources for later semantic integration processes. This conflict likely contributed to the attenuation of P200 amplitudes. Conversely, contextually congruent nouns aligned with expectations, facilitating perceptual matching and yielding larger P200 amplitudes. Notably, participants demonstrated sensitivity to musical regularity even when chords were task-irrelevant. Irregular chords elicited reduced P200 amplitudes over anterior and central regions compared to regular chords, which consistently produced larger amplitudes. Together, these findings indicate that domain-general attentional mechanisms mediate early language and music processing through top-down modulation of bottom-up perceptual analysis [62]. Specifically, contextual expectations (whether linguistic or musical) shape attentional resource distribution during initial sensory analysis, with violations prompting the reallocation of resources to downstream integration.

Importantly, our data revealed a significant cross-domain interaction between linguistic and musical processing during this stage. This interaction suggests overlapping neural mechanisms subserving initial processing in both domains. Specifically, over the midline and lateral sites, unacceptable sentences paired with irregular chords elicited smaller ERP amplitudes compared to the regular condition, while irregular musical contexts attenuated amplitudes more than in unacceptable sentences relative to acceptable ones. These findings imply that concurrent violations across domains (e.g., semantic incongruity in language and harmonic irregularity in music) compete for limited cognitive resources, resulting in diminished neural responses [7,63,64]. The amplitude reductions likely reflect heightened cognitive load under dual-violation conditions, where processing music-syntactic irregularities diverts resources from linguistic integration. This shared mechanism may represent the dynamic reallocation of resources, prioritizing subsequent integration stages and enabling the coherent unification of linguistic or musical elements into structured perceptual representations.

Additionally, the present results revealed hemispheric differences across central regions, with significantly larger negative ERP amplitudes observed over the right hemisphere compared to the left. This asymmetry aligns with the Early Right Anterior Negativity (ERAN), a component hypothesized to reflect music-syntactic processing based on tonal regularity between 150–300 ms post-stimulus [65]. This negativity might suggest that participants not only detected differences in musical regularity but also actively engaged in syntactic-level analysis of chord sequences. The presentation of irregular chords—those deviating from the established tonal framework—resisted integration with the preceding musical context, thereby eliciting the characteristic ERAN response.

### 4.2. The Underlying Shared Mechanisms at the Late Stage

Consistent with predictions, sentences ending with unacceptable nouns elicited larger N400 amplitudes during the late stage compared to acceptable endings. This N400 effect indicates active semantic integration between classifiers and sentence-final nouns, aligning with prior evidence that native Mandarin speakers process classifier–noun collocations through semantic expectancy [66,67]. To illustrate, classifiers such as “部 (bu)”, typically associated with electrical appliances, establish strong semantic expectations for semantically acceptable nouns [e.g., 手机 (shouji in Chinese pinyin, meaning “cell phone”)]. Unacceptable nouns [e.g., 钱包 (qianbao in Chinese pinyin, meaning “wallet”)] violate the prediction, increasing semantic integration demands and amplifying amplitudes of N400 responses. Furthermore, participants also demonstrated implicit sensitivity to musical regularity: irregular chords elicited more negative ERP amplitudes than regular chords. This suggests that despite being task-irrelevant, harmonic irregularities engaged in musical meaning integration processing [68]. Prior studies reported analogous N500 components, which are triggered by irregular chords or notes, reflecting challenges in musical meaning integration or the establishment of harmonic context [18,65]. This plausibly explains our observed neural profile: irregular chords elicited more negative ERP amplitudes than regular chords. Our findings suggest that parallel semantic integration processes occurred during this stage, with participants processing meaning integration for both task-relevant Chinese sentences and task-irrelevant chord sequences simultaneously.

Critically, during this time window, musical regularity modulated the processing of linguistic semantic acceptability. For both musicians and non-musicians, N400 amplitudes elicited by unacceptable nouns were influenced by harmonic regularity, indicating shared neural mechanisms for cross-domain semantic integration during the N400 latency [18,30]. However, this modulation occurred exclusively in semantically unacceptable sentences and was absent in acceptable conditions. This asymmetry likely reflects differential cognitive resource demands during real-time processing. Semantic integration of unacceptable nouns inherently requires more cognitive resources than for acceptable nouns, as reflected in enhanced N400 amplitudes. Similarly, integrating irregular chords, which violate harmonic expectations, requires greater effort than integrating regular chords, as evidenced by increased N500 amplitudes. When concurrent cross-domain violations occur (e.g., incongruent nouns paired with irregular chords), semantic integration processes compete for limited neural resources. This cross-domain competition amplifies integration demands while reducing resource availability for either linguistic or musical processing, manifesting neurophysiologically as attenuated ERP amplitudes (e.g., N400/N500 reductions). In contrast, acceptable sentence processing operates within baseline resource parameters, allowing for parallel analysis of musical stimuli without competitive interference through residual capacity allocation.

However, for non-musicians, the N400 amplitude at the midline in response to regular chords was only marginally significantly larger than that for irregular chords in the context of unacceptable sentences (*p* = 0.071). This suggests that non-musicians had limited cognitive resources available for processing musical regularity. Since musical regularity was task-irrelevant, non-musicians likely allocated most of their cognitive resources to sentence comprehension, leaving insufficient resources for processing the musical information. Moreover, the long-distance dependency between the classifier and head noun in these sentences may have imposed a high processing demand. If the semantic integration difficulty were reduced—for example, by shortening the distance between the classifier and head noun—this effect might reach statistical significance. Further empirical evidence is needed to test this possibility.

Musical stimuli also demonstrated a cross-domain modulating effect on linguistic processing within the P600 latency window. While the P600 component is traditionally linked to syntactic reanalysis or repair processes [69,70], the absence of syntactic violations in our stimuli precludes this interpretation. Instead, following Hsu et al. [71], the P600 responses to classifier–noun mismatches likely reflect the cognitive costs associated with updating mental models during long-distance dependency resolution. Meanwhile, irregular chords, defined as those deviating by more than three steps from the tonic within the tonal context based on the circle of fifths, appear to engage comparable harmonic integration in music sequences. Specifically, both linguistic and musical integration processes appear to involve distance-sensitive mechanisms, creating cross-domain interference. Unacceptable head nouns impose greater integration demands when establishing long-distance dependencies with classifiers. Similarly, irregular chords present heightened integration challenges within established tonal contexts. This parallelism reveals concurrent, distance-sensitive mental model updating across domains—linguistic processing requires dynamic reconfiguration of classifier–noun dependencies, whereas musical integration involves revising tonal context proportional to chord-key distance. Such cross-domain parallelism suggests shared neurocognitive mechanisms for semantic integration that transcend modality-specific features. Rather than simply overlapping in discrete processing characteristics, language and music appear to recruit domain-general resource allocation systems to resolve long-distance dependencies and establish semantic coherence. This aligns with Slevc and Okada’s [32] framework of shared neural architectures for complex sequence processing.

We would like to note that in the irregular condition, non-musicians exhibited only a marginally significant increase in P600 amplitude for unacceptable sentences compared to acceptable sentences (*p* = 0.058). Behavioral data revealed high accuracy (>80%) across conditions, with no significant difference between judgments of acceptable and unacceptable sentences, consistent with the ERP findings. These results suggest that sentence processing in non-musicians is modulated by musical regularity. As previously discussed, the P600 component is hypothesized to reflect mental model updating, the marginal significance observed here may imply limited cognitive resources allocated to sentence reading in non-musicians, thereby constraining their capacity to update mental representations in response to violations. We speculate that clearer effects might emerge under reduced sentence complexity (e.g., short-distance classifier–noun relationships). This hypothesis, however, requires empirical validation through targeted experiments.

### 4.3. The Potential Modulation of Shared Mechanisms by Musical Expertise

Our results systematically demonstrate music-mediated modulatory effects on linguistic semantic processing across temporal dynamics of concurrent language and music processing, revealing partially overlapping neurocognitive mechanisms for cross-domain meaning integration. Intriguingly, musicians and non-musicians exhibited divergent processing patterns during the later stage, characterized by opposing amplitude modulations of N400 and P600 components in response to semantically unacceptable head nouns. Non-musicians showed enhanced N400 amplitudes coupled with reduced P600 amplitudes following exposure to irregular chords. In contrast, musicians displayed the inverse pattern, with attenuated N400 amplitudes and amplified P600 amplitudes under the same conditions. This double dissociation suggests that musical expertise fundamentally restructures the shared neural infrastructure underlying language-music semantic integration, with distinct cross-domain compensatory strategies employed during semantic reprocessing based on musical expertise.

In the early stage, ERP responses showed no significant between-group differences, suggesting that musicians and non-musicians employed similar processing strategies. Both groups likely allocated comparable attentional resources to task-irrelevant musical stimuli or engaged in equivalent early semantic integration mechanisms. This implies that musical expertise does not modulate initial cross-domain meaning processing. However, this observation appears inconsistent with established theoretical frameworks. According to the OPERA hypothesis [72], musical training enhances domain-specific perceptual acuity that subsequently refines neural circuitry crucial for linguistic processing. Supporting this, musicians typically demonstrate superior attentional control and apply explicit musical knowledge when processing chord progressions, as studies have shown that irregular chords elicit an ERAN component in musicians during early processing stages [73], a neural signature of automatic music-syntactic violation detection that is absent in non-musicians [10,74]. This discrepancy suggests that long-term musical training may strengthen musical representations through procedural learning mechanisms, potentially leading to more automated early processing [75], without necessarily altering initial cross-domain interaction patterns.

The absence of early-stage differences in this study may be attributed to the experimental design. While participants were concurrently presented with linguistic and musical stimuli, explicit instructions emphasized semantic judgments, likely leading both groups to allocate cognitive resources predominantly to linguistic analysis. Cross-modal integration may thus have been deferred to later stages after initial syntactic violations were resolved. This strategic resource prioritization could explain both the lack of expertise-dependent early neural divergence and the subsequent group differences observed during later processing.

Notably, late-stage ERP responses revealed a double dissociation between the groups. Non-musicians displayed significantly amplified N400 amplitudes accompanied by attenuated P600 responses to harmonically irregular chords when reading unacceptable sentences. In contrast, musicians showed reduced N400 amplitudes and enhanced P600 components under identical conditions. This double dissociation may suggest group-specific neurocognitive mechanisms within shared language-music processing pathways.

On one hand, musical training appears to directly modulate neural responses to harmonic irregularities, as evidenced by expertise-dependent ERP responses. For instance, Featherstone et al. [76] proposed that musicians, through long-term training, develop an analytic listening strategy that integrates harmonic incongruities into the existing musical context, eliciting a P600 effect. Whereas non-musicians, lacking formal knowledge of musical regularities, rely on more holistic processing for harmonic integration, manifesting an N500 response.

In the present study, N400 amplitude variations across musical regularity conditions indicate that both musicians and non-musicians detected harmonic anomalies when out-of-key chords were presented. Non-musicians likely perceived these anomalies as a general sense of dissonance rather than having explicit syntactic awareness, consistent with the N500 pattern. Concurrently, semantically incongruous nouns paired with the chords elicited N400 effects, reflecting increased semantic integration demands. The overlapping neural substrates for semantic (N400) and harmonic (N500) integration may have interacted synergistically: shared negative polarity between N400 and N500 components likely amplified the N400 amplitudes, while the opposing polarity of the P600 component contributed to its suppression. However, musicians not only detected harmonic violations but also used their syntactic expertise to resolve irregularities within the musical framework, employing a more localized and analytical processing approach than non-musicians. This top-down integration process attenuated the N400 (by resolving semantic-harmonic conflicts) while enhancing the P600 amplitude, reflecting their capacity for hierarchical integration of harmonic structure.

Another plausible explanation for the divergent ERP variation patterns between groups lies in the gradual optimization and integration of domain-specific processing mechanisms shaped by musical expertise. Behaviorally, non-musicians showed sensitivity solely to linguistic semantics, with musical regularity exerting no significant influence on their judgments. In contrast, musicians exhibited sensitivity to both linguistic congruency and musical regularity, showing facilitation effects when linguistic and musical stimuli were either congruent or incongruent. That is, in the simultaneous presentation paradigm, the processing mechanisms between language and music were modulated by musical expertise, resulting in divergent shared processing patterns between the two groups.

This perspective aligns with competing hypotheses that explain the overlap between language and music processing. The neural overlap hypothesis posits that language and music recruit overlapping brain regions with independent but functionally analogous mechanisms [77]. It is theorized that neural circuitry originally dedicated to musicality evolved to support linguistic processing [31,78]. Within this framework, when language and music undergo parallel domain-specific integration processes, their independent but homologous mechanisms may interact, amplifying neural responses and producing facilitation effects [79,80]. This hypothesis is consistent with the behavioral and ERP profiles of non-musicians in the current study. Specifically, during the N400 time window, amplitudes for semantically unacceptable sentences paired with irregular chords were significantly larger than those paired with regular chords. This implies that concurrent integration of musical and linguistic meaning engaged distinct but interacting mechanisms, with musical processing enhancing linguistic semantic analysis and amplifying the N400 amplitudes—a pattern corroborated by prior studies [30,80].

On the other hand, the neural sharing hypothesis argues that language and music not only overlap in cortical regions but also compete for the same neural resources [80]. This competition may reduce the cognitive resources available for domain-specific processing, offering a plausible explanation for musicians’ ERP results: irregular chords paired with unacceptable sentences elicited significantly smaller N400 amplitudes than those paired with regular chords. Under this framework, cognitive resources typically allocated to linguistic semantic integration were partially reallocated to resolving musical harmonic regularities, which is essentially the simultaneous musical meaning integration, thereby attenuating the N400 amplitude for linguistic processing. Consequently, with increasing musical expertise, musicians develop enhanced domain-general cognitive control abilities, enabling the streamlined cross-domain encoding of semantic integration through statistical learning. Over time, the initially independent processing mechanisms for language and music may converge into a unified system optimized for cross-domain semantic integration. To sum up, these findings suggest that the shared neurocognitive mechanisms underlying language-music processing differ qualitatively between groups. Non-musicians appear to rely on distinct but interacting domain-specific mechanisms for language and music processing, whereas musicians, through long-term instrumental training, may consolidate these initially separate mechanisms into a unified processing system. This consolidation reflects adaptive neural plasticity, allowing musicians to integrate cross-domain regularities more efficiently through specialized superior cognitive control abilities.

Our findings demonstrate that Mandarin Chinese linguistic processing and musical sequences share neural resources during both the early and late stages of semantic processing, with musical expertise exerting modulatory effects on these shared mechanisms to enhance cross-domain processing efficiency. These results not only reveal how long-term training reshapes domain-general cognitive architectures but also highlight the translational potential of music-based interventions to amplify neuroplasticity in clinical populations (e.g., aphasia rehabilitation) and to optimize cognitive control in aging or language-learning contexts through cross-domain cognitive priming.

## 5. Conclusions

This EEG study investigated whether Mandarin Chinese and chord sequences share common semantic processing mechanisms, and how musical expertise modulates the underlying shared mechanism. Our findings provide novel evidence that Mandarin Chinese and chord sequences rely on partially overlapping neural mechanisms for semantic integration, with cross-domain effects emerging at the late processing stages (N400 and P600). Crucially, this represents the first demonstration that musical expertise fundamentally reshapes the shared mechanism. Specifically, musicians exhibited attenuated N400 responses coupled with enhanced P600 components, whereas non-musicians displayed the inverse pattern. These findings suggested that long-term music training may induce neuroplastic adaptations, structuring how the brain processes cross-domain semantic relationships. Furthermore, superior domain-general cognitive control abilities—cultivated through sustained musical practice—appear to reorganize the interaction between linguistic and musical processing. Over time, this may drive the gradual integration of two initially independent but functionally analogous mechanisms (linguistic and musical semantic integration) into a unified, domain-general system. Such plasticity underscores the dynamic interplay between experience-dependent learning and the brain’s capacity to optimize shared neural resources for multimodal tasks.

The broader implications of these results are multifaceted. For language learning, our findings highlight the potential for musical training to support the development of robust semantic networks, suggesting that music education could be a valuable complement to language acquisition programs. Similarly, in cognitive training, integrating music-based interventions might enhance domain-general cognitive control abilities, leading to more efficient allocation of neural resources. These insights pave the way for novel interdisciplinary approaches in educational settings and therapeutic interventions (e.g., aphasia rehabilitation).

Several limitations of this study should be acknowledged. First, the ERP differences observed are correlational and do not establish a direct causal link with musical training. The inherent spatial resolution limits of EEG could have constrained the precision of our neural measurements. Moreover, the lack of a detailed assessment of musical expertise may restrict the generalizability of the findings. Additionally, while Mandarin Chinese relies on pitch variations to distinguish lexical meaning, our study did not investigate how tonal processing interacts with shared semantic representations. Finally, since the comprehension of language and music is influenced by cultural background, our study did not address the impact of culture on the processing of language and music.

Future research should explore: (1) employing longitudinal fMRI to track neural plasticity during skill acquisition or using experimental designs (e.g., short-term musical training interventions) to directly test how training shapes cross-domain integration; (2) conducting a more nuanced assessment of musical expertise by categorizing participants based on specific proficiency levels, training durations, or types of musical experience; (3) examining the influence of pitch variations in tonal languages on semantic processing; and (4) including diverse musical genres that represent various cultural backgrounds.

## Figures and Tables

**Table 1:** Individual difference measures for musicians and non-musicians.

	Musicians		Non-Musicians		*t*-Test
	Mean	SD	Mean	SD	
age	21.73	1.67	21.83	1.69	0.214
Years of Education	15.83	1.69	15.72	1.67	0.132
Vocabulary	71.45	4.30	69.92	3.67	−1.324
MBEA (%)	94.87	2.29	98.09	1.30	−5.802 ***
Years of musical training	13.23	2.25	-	-	-
Onset of musical training	8.50	1.54	-	-	-

Note: *** denotes *p* < 0.001.

**Table 2:** Examples of experimental sentences.

Condition	Exemplar Sentence							
Acceptable	警察	捡到	了	一部	游客	丢失	的	手机
	The policeman	picked	LE (a perfective aspect marker)	one BU (CL: classifying electric appliance)	tourist	lost	DE (a modification marker)	cell phone
	The policeman picked up a cell phone, which might have been left by a tourist.							
Unacceptable	警察	捡到	了	一部	游客	丢失	的	钱包
	The policeman	picked	LE (a perfective aspect marker)	one BU (CL: classifying electric appliance)	tourist	lost	DE (a modification marker)	wallet
	The policeman picked up a wallet, which might have been left by a tourist.							

## Data Availability

The data are not publicly available due to the Informed Consent Form states that the data access is restricted to authorized researchers only.
